# Clinical value of neuroimaging indicators of intracranial hypertension in patients with cerebral venous thrombosis

**DOI:** 10.1007/s00234-024-03363-6

**Published:** 2024-04-27

**Authors:** Florian F. Schuchardt, Niklas Lützen, Sebastian Küchlin, Michael Reich, Wolf A. Lagrèze, Hansjörg Mast, Matthias Weigel, Stephan Meckel, Horst Urbach, Cornelius Weiller, Andreas Harloff, Theo Demerath

**Affiliations:** 1 Department of Neurology and Neurophysiology, Medical Center – University of Freiburg, Faculty of Medicine, University of Freiburg, Freiburg, Germany; 2 Department of Neuroradiology, Medical Center – University of Freiburg, Faculty of Medicine, University of Freiburg, Freiburg, Germany; 3 Eye Center, Medical Center – University of Freiburg, Faculty of Medicine, University of Freiburg, Freiburg, Germany; 4 Translational Imaging in Neurology (ThINk) Basel, Department of Biomedical Engineering, Faculty of Medicine, University Hospital Basel and University of Basel, Allschwil, Switzerland; 5 Department of Neurology, University Hospital Basel, Basel, Switzerland; 6 Research Center for Clinical Neuroimmunology and Neuroscience Basel (RC2NB), University Hospital Basel and University of Basel, Basel, Switzerland; 7 Division of Radiological Physics, Department of Radiology, University Hospital Basel, Basel, Switzerland; 8 Institute of Diagnostic and Interventional Neuroradiology, RKH Kliniken Ludwigsburg, Ludwigsburg, Germany

**Keywords:** Cerebral venous thrombosis, Intracranial hypertension, Neuroimaging indicators, Optic nerve sheath diameter, Partially empty sella

## Abstract

**Purpose:**

Intracranial hypertension (IH) frequently complicates cerebral venous thrombosis (CVT). Distinct neuroimaging findings are associated with IH, yet their discriminative power, reversibility and factors favoring normalization in prospective CVT patients are unknown. We determined test performance measures of neuroimaging signs in acute CVT patients, their longitudinal change under anticoagulation, association with IH at baseline and with recanalization at follow-up.

**Methods:**

We included 26 consecutive acute CVT patients and 26 healthy controls. Patients were classified as having IH based on CSF pressure > 25 cmH_2_O and/or papilledema on ophthalmological examination or ocular MRI. We assessed optic nerve sheath diameter (ONSD), optic nerve tortuousity, bulbar flattening, lateral and IV^th^ ventricle size, pituitary configuration at baseline and follow-up, and their association with IH and venous recanalization.

**Results:**

46% of CVT patients had IH. ONSD enlargement > 5.8 mm, optic nerve tortuousity and pituitary grade ≥ III had highest sensitivity, ocular bulb flattening and pituitary grade ≥ III highest specificity for IH. Only ONSD reliably discriminated IH at baseline. Recanalization was significantly associated with regressive ONSD and pituitary grade. Other neuroimaging signs tended to regress with recanalization. After treatment, 184.9 ± 44.7 days after diagnosis, bulbar flattening resolved, whereas compared with controls ONSD enlargement (*p* < 0.001) and partially empty sella (*p* = 0.017), among other indicators, persisted.

**Conclusion:**

ONSD and pituitary grading have a high diagnostic value in diagnosing and monitoring CVT-associated IH. Given their limited sensitivity during early CVT and potentially persistent alterations following IH, neuroimaging indicators can neither replace CSF pressure measurement in diagnosing IH, nor determine the duration of anticoagulation.

**Supplementary Information:**

The online version contains supplementary material available at 10.1007/s00234-024-03363-6.

## Introduction

Intracranial hypertension (IH) frequently complicates cerebral venous thrombosis (CVT). It occurrs early in around 40% [1–3] and as a long-term complication in about 10% of patients [4]. IH is associated with visual loss [5], long-term morbidity and mortality [6]. Even in regressive thrombosis delayed IH may occur [3, 5, 7]. Obvious reasons for elevated intracranial pressure comprise space occupying hemorrhage, edema, or venous infarction. Risk factors for IH in patients without intracranial mass lesions include thrombosis of dominant and bilateral transverse sinus [3, 8, 9], superficial venous thrombosis [3, 10], and collateralization connecting dural sinus with deep intracranial veins [11].

IH, both symptomatic and subclinical, threatens vision and the extent of visual impairment is associated with cerebrospinal fluid (CSF) pressure [5]. Diagnosing CVT-related IH requires invasive CSF pressure measurement [12]. Yet, anticoagulation must be paused to perform a spinal tap, potentially causing recurrent and progressive thrombosis. Papilledema is a useful 95–100% specific surrogate for IH [13, 14], applicable for non-invasive screening. But even without papilledema CVT frequently causes retinal damage [15].

**Fig. 1:**
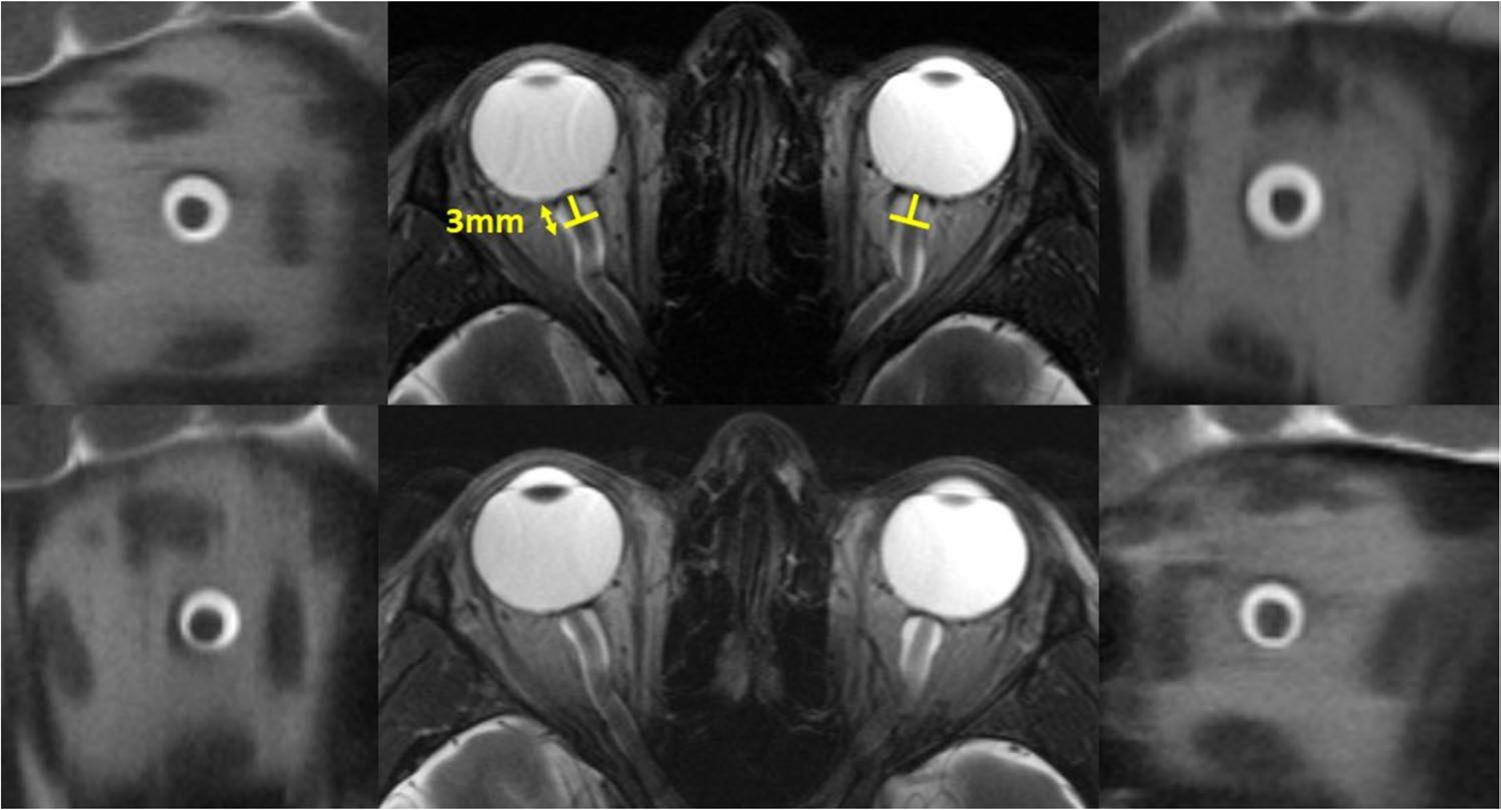
Reduction of the optic nerve sheath diameter between baseline (upper row) and follow-up (lower row) of patient P7, who presented with intracranial hypertension at diagnosis. The yellow bars in the upper central axial image show the position of 2D cross-sections 3 mm behind the optic nerve head, transsecting the right and left optic nerve perpendicular to its longitudinal orientation. The adjacent coronal images depict the cerebro-spinal fluid around the optic nerve in its sheath within the orbit, abating between baseline and follow-up imaging after 6 months. Also note the optic nerve tortuousity (upper and lower axial images)

In addition to elevated CSF pressure and papilledema, several MR imaging signs are associated with IH (see Fig. 1, supplementary Figs. 1–4). Ocular signs include increased optic nerve sheath diameter (ONSD), optic disc protrusion, posterior scleral flattening, and vertical optic nerve tortuosity. Parenchymal signs include cerebral ventricle size changes and decreased pituitary height [14, 16–18]. Most are assessable using standard MRI sequences or by easily implementable non-routine sequences, such as ONSD, which is also readily examinable using bedside transbulbar sonography [19]. A strong association of these neuroimaging findings with IH was retrospectively determined in selected CVT cohorts with elevated CSF pressure [14, 16].

The discriminative power of these neuromaging findings, yet, has not been established in prospective, unselected acute CVT patients. In addition, we have little information about the reversibility of neuroimaging signs during treatment and factors favoring their regression. Thus, IH neuroimaging indicators persisting after CVT may cause uncertainty regarding the duration of oral anticoagulation and the necessity of invasive diagnostics, such as lumbar puncture (LP).

We assessed test performance measures (sensitivity, specificity, positive and negative predictive values) of IH-associated neuroimaging surrogates during acute CVT. We compared patients with IH versus patients without IH to controls. IH was defined by a compound classifier consisting of CSF pressure > 25 cmH_2_O [12], papilledema on ophthalmological examination and/or optic disc protrusion on MRI [3]. In addition, we analyzed the reversibility of neuroimaging findings under anticoagulation, their association with IH at baseline and with venous recanalization at follow-up.

## Methods

### Cohort

Between January 2012 and December 2017, we prospectively registered 70 patients presenting with acute CVT to our institution. Inclusion criteria were age > 18 years and CVT diagnosed by neuroimaging (CCT and CT-Angiography or MRI and MR-Angiography). Of these, 26 patients who completed the MRI study protocol were included in the cohort (supplementary Fig. 5). 26 age- and sex-matched healthy volunteers without neurological disease underwent the study MRI to serve as controls.

General exclusion criteria were contraindications against MRI (ferro-magnetic implant, pregnancy, claustrophobia), critical illness (e.g. mechanical ventilation, severely altered consciousness), preexisting conditions causing intracranial hypertension (idiopathic intracranial hypertension, hydrocephalus) and denial to participate. We further excluded CVT patients showing space occupying hemorrhage, edema or venous infarction with midline-shift or lateral ventricle compression (see below) [3] and patients with symptoms suggestive of IH, in whom CSF pressure measurement, ophthalmological examination and/or ocular MRI were unavailable.

After diagnosing CVT, patients underwent cerebral and ocular MRI and, upon clinical suspicion of IH, CSF pressure measurement and additional ophthalmologic examination.

All 26 patients were part of a previous cross-sectional assessment of IH predictors in acute CVT [3].

### Intracranial hypertension

We classified CVT patients as having intracranial hypertension based on the following predefined criteria [3]:

- Symptoms of acute CVT (e.g. headache, seizure [20]) and
- intracranial pressure > 25 cm H_2_O (according to ICHD-3 classification [12]) measured by CSF pressure in lateral decubitus position and/or
- papilledema [13] on optical coherence tomography and/or optic disc protrusion on ocular MRI

As described before [3], we chose papilledema for its 95–100% specificity for IH [13, 14]. Given reports of unilateral papilledema in IH, we rated both uni- and bilateral optic nerve head protrusion as an indicator for IH [21]. Patients were defined as IH negative when they lacked criteria of the compound classifier.

### MR-Imaging

CVT patients underwent baseline cerebral MRI at inclusion (3 Tesla MRI, Magnetom TRIO or 1.5 Tesla MRI, Magnetom Avanto, Siemens Healthcare, Erlangen, Germany) including 2D T1, 2D T2, 2D time-of-flight (TOF) venography, 3D fluid attenuated inversion recovery based on sampling perfection with application-optimized contrasts using different flip angle evolution (FLAIR SPACE), 2D susceptibility-weighted imaging (SWI), and 2D high-resolution T2-weighted half-Fourier acquisition single-shot turbo spin-echo (HASTE) sequences as previously described [19, 22, 23]. Supplementary Table 1 gives sequence details. We complemented dedicated orbital imaging and contrast-enhanced (ce-) vascular imaging at 3 T soon after CVT diagnosis, as described before [3]. Controls underwent the same MR protocol at 3 T as patients, yet without application of Gd contrast agent. CVT follow-up comprised the same MR protocol and neurological examination.

### Venography

If not contraindicated, we acquired ce-T1W magnetization-prepared rapid acquisition with gradient echo (MPRAGE; supplementary Table 1) venography after acquision of the other sequences, approximately 4 min. after intravenous injection of 0.1 mmol/kg Gadoteridol (ProHance, Bracco Imaging, Milan, Italy), available in 24 patients. In two patients, we analyzed ce-CT-venography scans acquired after intravenous injection of 70 ml Iomeprol (Imeron 400, Bracco Imaging, Milan, Italy) at a flowrate of 3 ml/s, followed by 60 ml saline. CT-venography was started manually with inflow of contrast agent into the internal jugular vein using visual bolus tracking, as described before [3] using Siemens Somatom Definition AS and Definition Flash CT scanners (Siemens Healthcare, Erlangen, Germany); tube voltage 100 kV, exposure 140 mAs, collimation of 64 × 0.6 mm, helical mode, reconstruction of 0.75 mm isotropic datasets.

### Blinded assessment

MRIs were rated by four experts with longstanding experience in neuroimaging analysis (blinded readers: TD and NL > 10 years, SM > 20 years in neuroradiology; FS > 10 years in cerebral MRI research) who interpreted anonymized data using standard imaging software (IMPAX EE R20 DeepUnity Diagnost, Dedalus HealthCare GmbH, Bonn, Germany). TD and SM analyzed venographies blinded to clinical characteristics, complementary morphological data and rating performed by NL and FS and vice versa.

Two Neuroophthalmologists with > 10 years (MR) and > 25 years (WL) experience in ophthalmology performed funduscopy, being unblinded to the CVT diagnosis but unaware of MRI and LP related data.

Physicians performing lumbar puncture were blinded to ophthalmologic and neuroimaging findings.

### Presence of lesions

We identified CVT-associated cerebral edema in 3D FLAIR SPACE and hemorrhage using susceptibility-weighted imaging as described before [22]. In addition, on axial and coronal slices we searched for IH causes not attributable to thrombotic material, assessing any midline shift, lateral ventricle compression or dislocation of the temporal horns [3].

### Thrombosis rating

TD systematically determined the location and number of thrombosed intracranial veins and sinus on all available time-of-flight (TOF) and ce-MPRAGE venographies [24] as described before [3]. One point was assigned to each thrombosed part of the following 18 predefined segments (1–18 points): rostral, mid and dorsal segments of the superior sagittal sinus (SSS), transverse and sigmoid sinus, basal veins of Rosenthal, great vein of Galen, straight sinus and cortical veins draining into the SSS including the superior and inferior collaterals of Trolard and Labbé.

### Recanalization rating

TD and SM independently rated thrombus recanalization on follow-up ce-MRV as described before [3](supplementary Table 2), using the grading proposed by Aguiar de Sousa [25] and Qureshi [26]. Readers resolved disagreement by consensus. We analyzed the influence of recanalization extent (any degree, partial (i.e. Aguiar de Sousa grade ≥ 2A, see supplementary Table 3) and complete recanalization (Qureshi grade III, Aguiar de Sousa grade 3)).

### Neuroimaging indicators of intracranial hypertension

NL rated the presence of optic disc protrusion [3], bulbar flattening and optic nerve tortuousity [17] depicting the bulbi and optic nerves (Fig. 1; supplementary Fig. 1), pituitary tissue was graded using 3-dimensional FLAIR SPACE [22] according to Yuh et al. (grade 1, normal appearance through grade 5, empty sella; grade 3 representing abnormal partially empty sella; supplementary Fig. 2)[16]. Lateral and fourth ventricle size (supplementary Figs. 3 and 4) was determined using standardized measurements on axial T1w images as previously described [14].

FS analyzed ONSD (Fig. 1) and frequency of enlargement > 5.8 mm [18, 19] using HASTE-sequence as described before [19, 23]. In short, we placed a circular region-of-interest marker 3 mm behind the eyeball, perpendicular to longitudinal extension of the optic nerve. ONSD was defined as the border of highest contrast of signal intensity change between CSF and the surrounding optic nerve sheath.

### Statistics

Continuous data are given in mean ± standard deviation (SD). We assessed cross-sectional and longitudinal differences of continuous variables by simple and dependent t-test and categorical data by chi^2^ or Fishers’ test. We determined sensitivity and specificity, positive and negative predictive values (PPV, NPV) and odds ratios (OR) of neuroimaging signs. Ordinal variables were analyzed by Mann Whitney U (cross-sectional) or Wilcoxon rank sum tests (longitudinal). We determined associations of continuous, categorical and ordinal variables by Kendall’s tau.

All tests were two-sided if not stated otherwise, with an α-level of significance set at 5%. We considered missing data to occur at random and did not impute. *P*-values were not corrected for multiple testing and should be interpreted as hypothesis generating.

## Results

### Cohort

We included 26 CVT patients (21 female, 77.8%) and 26 age- and sex-matched controls. Patients’ demographics are presented in Table 1. Average time from CVT diagnosis to inclusion was 7.3 ± 8.7 days. 23 patients underwent follow-up ce-MRI after 184.9 ± 44.7 days, in the majority 5–7 months after CVT diagnosis. Later imaging was performed in three patients after 245 to 335 days. Of these, one patient (P7) was IH positive and two (P4 and P12) IH negative.

**Table 1:** Patients ‘ demographics

ID	Age [years]	Sex	Symptoms at presentation	IH classification	CSF pressure [cm H_2_O]	Papilledema on Fundoscopy	Optic disc protrusion on MRI	neuroimaging				Anticoagulation: acute, longterm; duration	Symptoms at follow up	mRS before CVT / at follow-up
								ONSD [mm]		Bulbar flattening	Pituitary grading			
								right	left					
P1	21	f	progressive headache, nausea, emesis	positive	42	n.a	absent	n.d	n.d	absent	1	Nadroparine, Phenprocoumone, 6 months	intermittent unilateral temporal headache, lasting minutes	0 / 1
P2	18	f	progressive cephalalgia, nausea, emesis, cranial nerve VI paresis; IH symptoms 3.5 weeks after onset of headache, 4 days after initiation of anticoagulation	positive	60	bilateral	n.a	6.4	n.d	present	3	unfractioned heparine, Phenprocoumone, 12 months	none	0 / 0
P3	23	f	generalized tonic–clonic seizure with fall, non-fluent aphasia	negative	n.d	absent	absent	n.d	n.d	absent	2	Nadroparine, Phenprocoumone; 12 months	none	0 / 0
P4	45	f	generalized tonic clonic seizure, postictual psychomotor slowing	negative	16	n.a	n.a	4.9	4.9	n.d	2	unfractioned heparine, Nadroparine, Phenprocoumone; 6 months	occasional concentration deficit	0 / 0
P5	63	f	aphasia, disorientation, somnolence	negative	n.d	n.a	absent	5.0	4.9	absent	2	unfractioned heparine, Nadroparine, Phenprocoumone; 6 months	non-fluent aphasia following aneurysmatic subarachnoid hemorrhage 1 month after CVT	0 / 2
P6	55	f	headache, diplopia, dysgeuisia	positive	50	bilateral	present	7.3	7.4	present	4	Nadroparine, Phenprocoumone for 5 weeks, change to Aspirine after subarachnoid hemorrhage	Diplopia in extreme lateral viewing direction	1 / 1
P7	28	m	headache	positive	50	bilateral	present	6.6	7.3	present	4	Nadroparine, Phenprocoumone; 6 months	none	0 / 0
P8	51	f	headache, aphasia	negative	15	n.a	absent	4.8	4.8	absent	4	unfractioned heparine, Phenprocoumone, continuous	deceased due to urothelial carcinoma	0 / 6
P9	18	f	headache, generalized tonic–clonic seizure, intubated at referral after seizure	positive	n.d	n.a	present	6.8	7.2	present	4	Nadroparine, Phenprocoumone; 12 months	none	0 / 0
P10	33	f	headache, nausea, emesis	negative	n.d	n.a	absent	6.3	5.6	present	2	Enoxaparine, Phenprocoumone; continuously	intermittent dyscognitive seizures, otherwise none	0 / 0
P11	22	m	headache, nausea, emesis	negative	n.d	n.a	absent	6.6	6.0	absent	2	Nadroparine, Phenprocoumone; 12 months	none	0 / 0
P12	33	f	headache, transient visual disturbance and aphasia	negative	18	n.a	absent	n.d	n.d	present	4	unfractioned heparine, Phenprocoumone, 18 months	none	0 / 0
P13	43	f	headache, clumsy right hand, tingling paresthesia	positive	27	absent at inclusion, n.a.at 5 month follow-up	absent	6.4	5.6	absent	3	Nadroparine, Phenprocoumone; 6 months	left hemihypaesthesia, secondary intracranial hypertension confirmed by CSF pressure measurement on day 192 after CVT diagnosis	0 / 1
P14	29	f	headache, dizziness	negative	n.d	n.a	absent	6.2	6.4	pres ent	3	Nadroparine, Phenprocoumone; 6 months	none	0 / 0
P15	40	m	headache, visual disturbance	positive	45	bilateral	absent	5.6	5.3	absent	3	Nadroparine, 7 months	slight visual disturbance	0 / 1
P16	32	m	headache, intermittent brachiofacial numbness	negative	n.d	n.a	absent	6.0	6.2	present	4	Enoxaparine, Phenprocoumone; 3 months	preexisting reduced vision of left eye	0 / 1
P17	47	f	trauma with parietal skull fracture and epidural hematoma	negative	n.d	n.a	absent	4.7	4.7	absent	1	Unfractioned heparine, phenprocoumone; 6 months	tinnitus	0 / 1
P18	20	f	headache, nausea, vomiting, phono- photophobia, diplopia	positive	38	n.a	n.a	n.d	n.d	n.d	4	Nadroparine, Phenprocoumone, 12 months	none	0 / 0
P19	56	f	generalized tonic–clonic seizure	negative	n.d	n.a	absent	6.5	7.0	present	3	Nadroparine, Phenprocoumone; 6 months	none	0 / 0
P20	26	f	somnolence, disorientation, hemispheric syndrome with aphasia, multimodal neglect, facial paresis, hemiparesis, hemihypaesthesia	negative	n.d	absent	absent	5.1	5.1	absent	2	Unfractioned heparine, Phenprocoumone; 6 months	none	0 / 0
P21	41	f	headache, emesis, lower quadrant hemianopia	positive	35	n.a	absent	6.4	6.7	present	2	Nadroparine, Phenprocoumone; 12 months	none	0 / 0
P22	50	m	headache	negative	n.d	absent	absent	6.3	5.8	absent	3	Unfractioned heparine, Dagibatran; stopped after 24 months after intermittent pulmonary embolisms	intermittent headache, persistent pulsatile tinnitus	0 / 1
P23	19	f	progressive headache, nausea, vomiting, blurred vision	positive	29	bilateral	present	7.4	7.1	present	3	Enoxaparine, Dabigatran, duration unknown due to external follow-up	lost to follow-up	0 / n.a
P24	18	f	headache, nausea	negative	n.d	n.a	absent	6.8	6.5	present	3	Enpxaparine, Dabigatran, 7 months	intermittent tension-type headache	0 / 1
P25	39	f	progressive headache for 2 months, troubled vision	positive	46	bilateral	present	9.1	9.2	present	3	Dabigatran, duration unknown due to external follow-up	lost to follow-up	1 / n.a
P26	21	m	progressive headache	positive	50	n.a	absent	n.d	n.d	absent	2	Nadroparine, Dabigatran, 12 months	none	0 / 0

*CSF* Cerebrospinal fluid; *CVT* Cerebral venous sinus thrombosis; *f* Female; *IH* Intracranial hypertension; *m* Male; *mRS* Modified Rankin scale; *N.a.* Not available; *n.d.* Not determined; *ONSD* Optic nerve sheath diameter

### Classification of intracranial hypertension

12/26 patients (46.2%) had IH (supplementary Fig. 6). We classified 14 patients by CSF pressure measurement (11 patients had elevated (42.9 ± 10 cmH_2_O) and three normal CSF pressure (16.3 ± 1.5 cmH_2_O)). Among 9 patients undergoing neuroophthalmologic examination, we classified three patients by the lack of papilledema (6 were already classified by CSF pressure). We classified 9 out of 23 patients by ocular imaging (one showing, 8 lacking papilledema; the remaining 14 patients were already classified by CSF pressure and ophthalmologic examination).

In this cohort, no patient had unilateral papilledema. Controls did not show papilledema at repeated MRIs 26.2 ± 10.9 days apart.

### Baseline imaging

#### Discriminative power of neuroimaging signs in CVT at baseline

For comparison with previously published retrospective data, Table 2 gives test performance metrics and odds ratios (OR) of neuroimaging findings. In patients with IH, ONSD enlargement > 5.8 mm, optic nerve tortuousity and partially empty sella (i.e. pituitary grade ≥ III) had highest sensitivity. Ocular bulb flattening, and pituitary grade ≥ III yielded highest specificity. NPV achieved at least 88.2%, whereas PPV were below 50%.

**Table 2:** Test performance of neuroimaging indicators of IH at baseline

Baseline	Sensitivity	Specificity	Positive predictive value	Negative predictive value	Odds ratio (95%CI)	
ONSD > 5.8 mm	88.9% (8/9)	61.5% (24/39)	34.8% (8/23)	96% (24/25)	With IH	16 (1.5–176.5)
					Without IH	5.9 (1.1–32)
Optic disc protrusion	50% (5/10)	n.a. (39/39)	n.a. (5/5)	88.6% (39/44)	With IH	n.a
					Without IH	n.a
Ocular bulb flattening	63.6% (7/11)	76.9% (30/39)	43.8% (7/16)	88.2% (30/34)	With IH	19.3 (1.8–209.6)
					Without IH	15.17 (1.5–152.5)
Optic nerve tortuousity	80% (8/10)	56.4% (22/39)	32% (8/25)	91.7% (22/24)	With IH	5.6 (0.8–38.5)
					Without IH	1.1 (0.3–5.2)
Pituitary grade ≥ III [16]	75% (9/12)	72.5% (29/40)	45% (9/20)	90.6% (29/32)	With IH	15 (2–111.2)
					Without IH	6 (1–37.3)

We classified CVT patients by intracranial hypertension classification (based on CSF pressure > 25 cm H_2_O and/or papilledema on ophthalmologic examination or ocular MRI) and compared neuroimaging findings to age- and sex-matched controls. *n.a.* Not applicable, being part of the IH classifier

Compared with controls, all neuroimaging signs had higher OR in patients with versus without IH. Yet, OR were also elevated in patients without IH compared to controls (in decreasing order: bulbar flattening, pituitary grade ≥ III, ONSD enlargement > 5.8 mm).

**Fig. 2:**
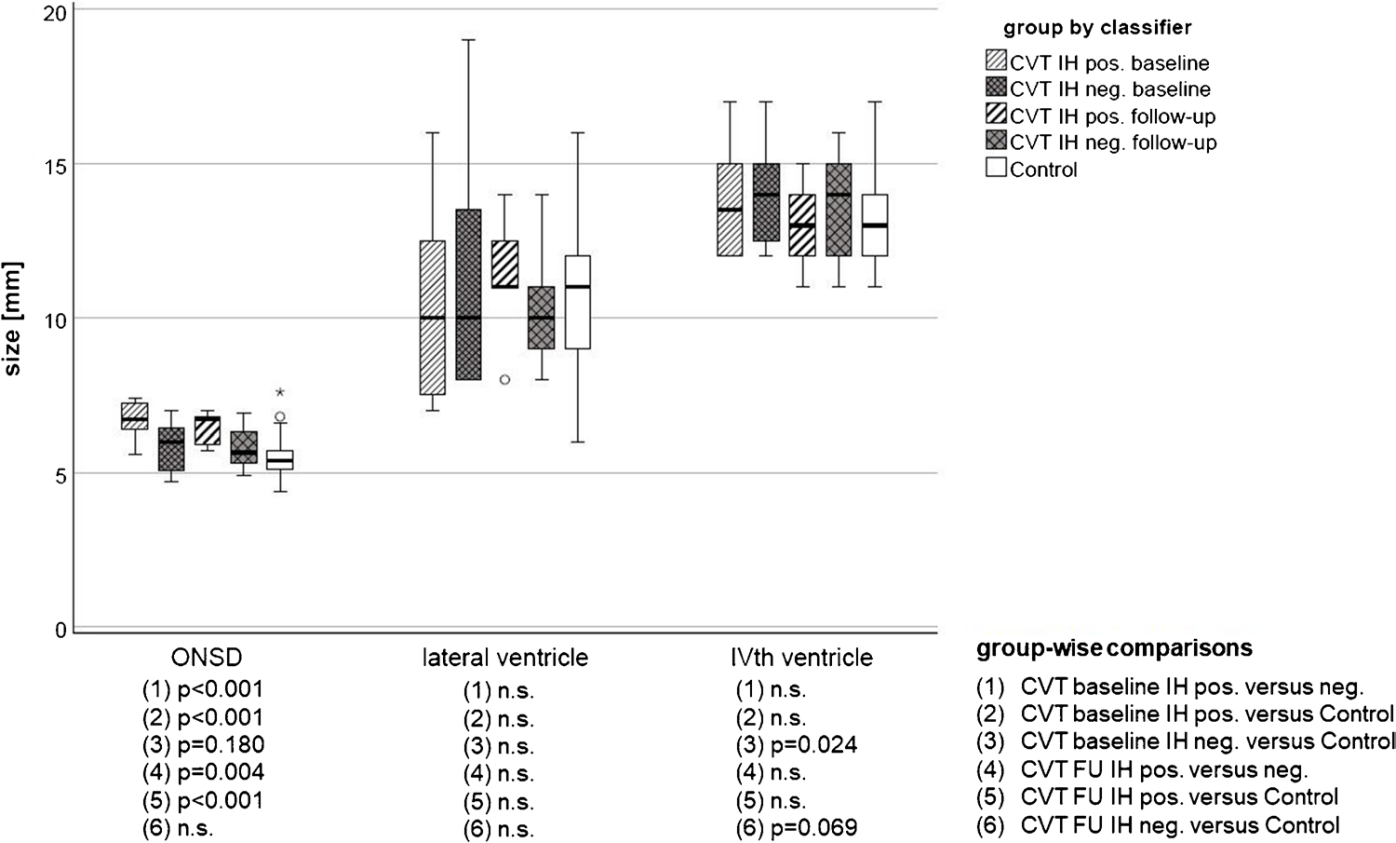
Boxplots show optic nerve sheath diameter (ONSD), lateral and IV^th^ ventricle size in CVT patients at baseline, follow-up (FU) and in normal controls. Comparisons at baseline give the discriminative potential of neuroimaging findings. Longitudinal comparisons demonstrate the extent of reversibility, depending on the classifier IH and in relation to normal dimensions in controls. In conclusion, only ONSD discriminated IH in CVT patients, and differed significantly at follow-up in patients with versus without IH and healthy controls. To improve readability, we omitted *p*-values > 0.2 (n.s., not significant)

Figure 2 shows the distribution of ONSD, lalteral and IV^th^ ventricle size in CVT patients compared with controls. Among ocular findings, significantly larger ONSD discriminated patients with IH from both patients without IH and controls (see Fig. 2; ONSD in patients without IH and controls were comparable).

At baseline, none of the other ocular neuroimaging signs (ocular bulb flattening, optic nerve tortuousity) nor the parenchymal findings (lateral ventricle size, IV^th^ ventricle size, and pituitary grade) discriminated CVT subgroups (Fig. 2, Table 2). Although at baseline IV^th^ ventricle size was significantly wider in CVT patients without IH than in controls, IV^th^ ventricle size in CVT patients with versus without IH were comparable. Pituitary grade discriminated controls from both patients with IH (*p* = 0.003) and patients without IH (*p* = 0.043). Yet, pituitary grade ≥ III did not discriminate between CVT subgroups (patients with versus without IH, *p* = 0.202; Mann–Whitney; one-sided comparisons in small sample), despite > 2.5-fold higher odds ratio in patients with IH (Table 2).

### Follow-up imaging

Until follow-up, IH symptoms resolved in most patients under treatment and greatly improved in P15 and P22 (Table 1). Patient P13 developed delayed IH, despite recanalization under anticoagulation (Aguiar de Sousa Grade 2B).

#### Reversibility of optic disc protrusion

Optic disc protrusion at follow-up was less frequent in patients with IH compared to baseline, without reaching statistical significance (baseline, 5/10 (50%); follow-up, 1/8 (12.5%), *p* = 0.152). No patient without IH developed optic disc protrusion until follow-up.

#### Reversibility of neuroimaging changes in CVT

**Fig. 3:**
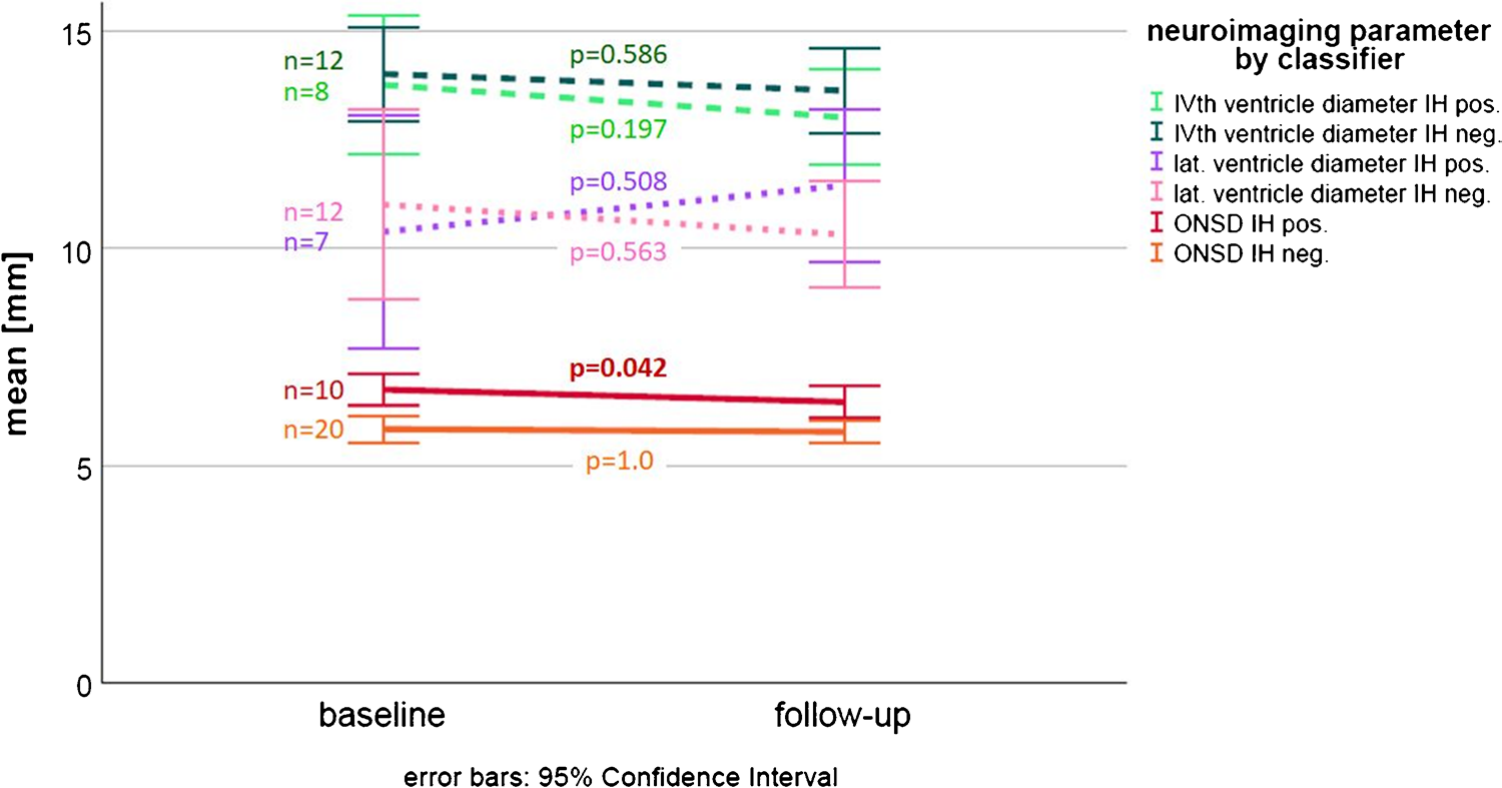
Line-plots show longitudinal within-group changes of IV^th^, lateral ventricle size, and optic nerve sheath diameter (ONSD) between baseline and follow-up classified by IH (intracranial hypertension). Numbers indicate available pairs

Patients with IH showed a significant ONSD reduction between baseline and follow-up (Fig. 3), whereas ONSD remained stable in patients without IH.

Longitudinal changes of the other neuroimaging findings including pituitary grade (patients with IH, *p* = 0.414; without IH, *p* = 0.317) did not reach significance (Fig. 3, Table 3), although bulbar flattening frequency halved in patients with IH and patients without IH showed a trend towards lower frequency.

**Table 3:** Change in prevalence of categorical imaging signs at baseline compared with follow-up in CVT patients by IH classification (based on CSF pressure > 25 cm H_2_O, and/or papilledema on ocular MRI or on ophthalmologic examination)

Longitudinal	IH	CVT baseline	CVT FU	Controls	*p*-value IH pos. versus neg. baseline	*p*-value baseline versus FU	*p*-value IH pos. versus neg. FU	*p*-value CVT FU versus matched Control
ONSD > 5.8 mm	Pos	8/9 (88.9%)	8/8 (100%)	4/12 (33.3%)	0.333	1.0	**0.046**	**0.005**
	Neg	8/13 (61.5%)	7/13 (53.8%)	3/14 (21.4%)		1.0		0.120
Ocular bulb flattening	Pos	7/11 (63.6%)	2/7 (28.6%)	1/12 (8.3%)	0.697	0.335	0.537	0.523
	Neg	7/13 (53.8%)	1/10 (10%)	1/14 (7.1%)		0.074		1.0
Optic nerve tortuousity	Pos	8/10 (80%)	6/8 (75%)	5/12 (41.7%)	0.197	1.0	0.642	0.197
	Neg	6/13 (46.2%)	7/12 (58.3%)	6/14 (42.9%)		0.695		0.695
Pituitary grade ≥ III [16]	Pos	9/12 (75%)	5/7 (71.4%)	2/12 (16.7%)	0.248	1.0	0.350	**0.045**
	Neg	7/14 (50%)	5/13 (38.5%)	2/14 (14.3%)		0.704		0.209

*FU* Follow-up

Statistically significant comparisons are highlighted in bold

#### Normalization of neuroimaging indicators of intracranial hypertension

Among ocular neuroimaging signs, ONSD size in patients with IH remained significantly larger at follow-up compared to patients without IH and controls (Fig. 2). In line, ONSD enlargement > 5.8 mm at follow-up remained significantly more frequent in patients with IH compared with controls, whereas frequency in patients without IH versus controls was comparable (Table 3). Bulbar flattening and optic nerve tortuousity were as frequent in patients with and without IH at follow-up as in controls.

Among parenchymal neuroimaging sings partially empty sella remained significantly more frequent in patients with IH compared to controls (pituitary grades, one-sided *p* = 0.017), contrasting comparable pituitary grades in patients without IH versus controls (one-sided *p* = 0.434). At follow-up, patients with IH showed non-significantly less visible pituitary tissue (i.e. higher pituitary grade) compared to patients without IH (Mann–Whitney, *p* = 0.091). Between-group differences of lateral ventricle size at follow-up, although larger in patients with IH than without IH, were comparable to controls (Fig. 2). Longitudinally, the IV^th^ ventricle showed stable dimensions in patients without IH, remaining larger than in controls (*p* = 0.033).

Taken together, in patients with IH, both absolute ONSD size, the frequency of ONSD enlargment > 5.8 mm and pituitary aspect did not normalize until follow-up, referring to controls. In IH negative patients, only the size of the IV^th^ ventricle (already significantly larger at baseline) remained larger at follow-up, compared to controls.

#### Association of neuroimaging findings ‘ reversibility with recanalization

Supplementary Table 2 shows aggregated frequencies of recanalization grades. Figure 3 shows longitudinal size differences of the individual imaging signs and supplementary Fig. 7 changes of ONSD of individual eyes by Aguiar de Sousa grade ≥ 2A (at least partial recanalization of all previously thrombosed vessel segments).

The neuroimaging findings yielding a significant correlation with at least partial recanalization of all thrombosed segments (Aguiar de Sousa grade ≥ 2A) in our cohort were ONSD size reduction (Kendall’s tau; 0.406, *p* = 0.01) and pituitary grade (2.2 ± 1 in those with lower versus 3.7 ± 0.5 with higher recanalization grades; Mann–Whitney, *p* = 0.05). In addition, longitudinal changes of ONSD and lateral ventricle size (Fig. 3) showed a non-significant correlation with different recanalization grades (supplementary Table 3). In contrast, CVT patients showing complete recanalization had comparable pituitary grade at baseline and follow-up (*p* = 0.317).

Further analyses regarding recanalization grade and IH did not reveal significant correlations.

## Discussion

In this prospective cohort including 26 patients with acute cerebral venous thrombosis (CVT), 12 (46%) had intracranial hypertension (IH), identified by CSF pressure > 25cmH_2_O and/or papilledema.

At baseline, ONSD was significantly larger and enlargement > 5.8 mm more frequent in CVT patients with versus without IH. Ocular bulb flattening and partially empty sella (pituitary grade ≥ III) had highest specificity, i.e. indicating low probability of IH when absent. Of high clinical value, negative predictive values of > 85% gave unremarkable findings in CVT patients classified not having IH. ONSD enlargement > 5.8 mm, optic nerve tortuousity and pituitary grade ≥ III showed highest sensitivity, supporting the diagnosis of IH. Neuroimaging indicators achieved positive predictive values below 50%, i.e. identified less than half of the patients classified having IH.

At follow-up, 70% of patients showed at least partial recanalization of previously thrombosed vessels, including 33% achieving complete recanalization. Reduction of ONSD and pituitary grade until follow-up were significantly associated with recanalization (at least partial recanalization of all previously thrombosed vessel segments; Aguiar de Sousa grade ≥ 2A). Despite significant regression, ONSD in patients with IH did not return to dimensions observed in patients without IH and controls. At follow-up, partially empty sella remained more frequent in patients with IH compared to controls, too, although all patients except for P13 showed marked improvement or resolution of IH symtoms.

### Causes of intracranial hypertension

Several often intertwined mechanisms increase the IH risk in CVT: First, the amount and localization of thrombus predispose to IH [3, 8, 9, 27]. Second, depending on collateralization, even local thrombosis may increase endovenous pressure. Third, the pressure dependend CSF resorption via glymphatic pathways is reduced by lower trans-vessel pressure e.g. when thrombosis involves Pacchioni granulations [28]. Fourth, although rare, dural arterio-venous fistula causing or complicating CVT may increase intravenous and intracranial pressure [29–31]. Fifth, local space occupation due to edema, infarction and hemorrhage following venous stasis may cause rise of intracranial pressure [32].

### Classification of intracranial hypertension

To classify IH, we relied on elevated CSF pressure [12] and if unavailable, on papilledema on ophthalmological examination and/or optic disc protrusion on MRI. The number of positive neuroimaging findings correlates with CSF pressure [33]. In addition, the prevalence of papilledema correlates with the number of IH neuroimaging indicators, increasing from 2.8% in patients presenting at least one sign to 40% in patients with ≥ 4 IH indicators [34].

With nearly one in two patients (46%) developing IH during acute CVT, IH prevalence was comparable to previous reports [1, 2]. IH rarely developed delayed [4], affecting two patients with progressive thrombosis 6 and 38 days after CVT diagnosis.

### Discriminative power

Our prospective CVT cohort appears closer to real-life in-hospital conditions than previous retrospective case–control cohorts selected by proven IH [14, 17], limiting their mutual comparability. Our results support the diagnostic value of ONSD enlargement regarding IH in acute CVT [18, 19]. In line with Dong et al. [14], pituitary grade differentiated CVT patients with IH and controls. The other neuroimaging indicators were less of avail. Although more common in patients with IH, optic nerve tortuousity had low sensitivity in identifying IH in acute CVT. Ocular bulb flattening frequency, pituitary grade, and—contrasting Dong et al. [14]—lateral and IV^th^ ventricle size did not discriminate CVT patients with versus without IH at baseline.

### Temporal course of morphological changes

Morphological changes may appear at different CVT stages and show a diverging dynamic. Given the 1–2 week latency from CVT diagnosis to inclusion in our cohort, we cannot exclude misclassification of patients who developed IH and associated morphological effects later. Despite elevated CSF pressure, five patients in our cohort did not show papilledema at inclusion. Papilledema in CVT mostly develops within one to two months [7]. The limited positive predictive value of neuroimaging findings might thus stem from the early CSF pressure measurement. In addition, the lack of papilledema in patients not undergoing CSF pressure measurement might have underestimated IH frequency.

### Reversibility of neuroimaging findings in CVT

Little data is available on the usefulness of neuroimaging findings as monitoring parameters during the later CVT course, e.g. to guide the duration of anticoagulation in case of incomplete recanalization. Reversibility of neuroimaging indicators appeared heterogeneous, more frequently occurring in ocular findings in our cohort. The frequency of ONSD enlargement, bulbar flattening and pituitary grade decreased significantly, and reversibility was associated with IH classification. Yet, despite significant regredience, ONSD enlargement did not regress to baseline values in 8/8 IH patients compared to controls and patients without IH. Pituitary grade even increased in patients with IH.

As IH symptoms resolved in most patients, we estimate ongoing IH at follow-up is less probable. Patient P13 developed delayed IH despite advanced recanalization. Showing markedly improving IH symptoms but lacking follow-up LP, we cannot exclude ongoing IH causing slight residual blurred vision in P15 and intermittent tinnitus in P22.

Duration of IH, tissue elasticity and the time needed for normalization might affect the remission of IH neuroimaging signs. In idiopathic intracranial hypertension (IIH) patients showing papilledema, MRI findings suggestive of IH persisted after resolution of both symptoms and papilledema [35]. In CVT, papilledema was reversible on average after 6 months [7]. In vivo intrathecal infusion tests [36] and post mortem experiments [37] demonstrate the interrelation between ONSD enlargment and short-term CSF pressure elevation. ONSD reversiblility was incomplete after high pressure exposure [37]. In our cohort, we ascribe the persistent ONSD enlargment to prolonged IH during acute CVT.

At follow-up, partially empty sella in patients with IH remained significantly more frequent compared with controls, however not reaching significance compared with CVT patients without IH. Although IH treatment might reverse empty sella [38], pituitary compression may outlast due to structural changes of the sellar region, as in IIH [35].

Regredience of bulbar flattening frequency did not differ in patients with versus without IH, potentially due to the limited cohort size. Longitudinal changes of inner CSF spaces and IH at baseline were not significantly associated in our cohort. In line, the size of inner CSF spaces neither predicted IH nor differed before versus one month after treatment of CVT [39].

### Association of reversibility of neuroimaging findings with recanalization

In the range of previous reports [40], depending on the grading used, 33% (Aguiar de Sousa) to 70% (Qureshi) showed complete and > 70% at least partial recanalization of all initially affected vessels. Several neuroimaging findings were non-significantly associated with partial recanalization of all previously thrombosed vessel segments (Aguiar de Sousa grade ≥ 2A). There was no correlation with presence or absence of complete recanalization alone.

Incomplete recanalization might predispose to longstanding IH, occurring in 10% after CVT [4]. In contrast, recanalization is associated with both normalization of hemodynamics, cerebral metabolism and positive outcome [25, 40–43]. Given variable collateralization networks, both the extent of recanalization necessary to prevent IH and the degree of venous flow restoration necessary for regredience of imaging alterations in CVT remain unclear.

### Strengths and limitations

Strengths of our study include the prospective CVT cohort, reflecting real-life in-hospital conditions, which we analyzed by dedicated, systematic rating of thrombosis and recanalization. We internally validated test performance measures of neuroimaging signs by comparing IH subgroups versus their separate matched controls. Our results offer an orientation regarding the evolution of neuroimaging findings after CVT.

Our study is limited by its size and monocentric design, and also by exclusion of patients lacking both CSF pressure measurement and ophthalmologic examination or ocular MRI. Second, our cohort possibly overrepresents patients with visual symptoms and, excluding critically ill patients, our results might not apply to severe CVT. Third, for ethical reasons, we renounced follow-up CSF pressure measurement to confirm IH reversibility. We think this is negligible as IH symptoms resoved in all patients. Only one patient (P9) showed asymptomatic optic disc protrusion on follow-up MRI. Our results need replication, ideally in a larger prospective cohort.

### Clinical consequences

The suspicion of CVT indicates emergent brain imaging work-up. Complementing ce-MR-venography, IH neuroimaging sings offer high specificity classifying IH. Unfortunately, the temporal course of IH and neuroimaging findings in CVT are highly variable and symptoms may precede morphological effects of IH. This carries the risk of misclassification and explains the limited positive predictive value of IH neuroimaging indicators. Thus, neuroimaging surrogates may support estimating the IH risk in CVT but appear insufficient to replace CSF pressure measurement.

Pausing anticoagulation for spinal tap carries the risk of progressive thrombosis. This requires evaluating the clinical presentation in synopsis with IH risk factors, e.g. thrombus location, extension and collateralization [3, 9, 11, 27]. Consequently, individual IH pretest probability should guide the decision to perform LP. Longitudinal ONSD assessment may be a helpful alternative, given the ease of sonographic bedside assessment.

Further, IH neuroimaging findings persisting after CVT or appearing delayed in asymptomatic patients may cause uncertainty regarding the need and duration of anticoagulation, clinical follow-up frequency and invasive CSF pressure measurement. In asymptomatic CVT patients, persisting IH indicators should promote non-invasive diagnostic steps before-hand, such as ophthalmological examination (e.g. optical coherence tomography, fundoscopy, ONSD sonography) [7, 13, 15]. In patients with a sustained suspicion of IH, we suggest determining CSF pressure to guide further therapy, e.g. continued anticoagulation or acetazolamide [44].

The optimal duration of anticoagulation is currently elusive [44] as 3/4 of patients recanalized within 3 months [40], but recanalization may occur as late as 800 days after CVT [42]. Therefore, patients showing incomplete recanalization need individualized counseling regarding anticoagulation duration, weighing the chances of further recanalization versus bleeding risk.

## Conclusion

Intracranial hypertension complicates acute CVT in nearly every second patient and may develop with delay. Therefore, the appearance of clinical signs or neuroimaging findings of IH and their reversibility warrant further investigation. In patients with acute CVT, neuroimaging features with high sensitivity for IH were ONSD enlargement, optic nerve tortuousity, and pituitary grade ≥ III (“partially empty sella”), supporting the diagnosis of IH. Lack of ocular bulb flattening and pituitary grade ≥ III had high specificity, indicating low IH probability. Finally, ONSD enlargement discriminated presence of IH.

Non-invasive ONSD measurement might guide the clinician in diagnosing and following up on CVT-related IH. In our study, ONSD and pituitary grade improved significantly, yet, did not reach dimensions observed in controls.

Taken together, neuroimagig indicators in synopsis with clinical presentation and IH risk factors should guide clinical reasoning and diagnostic procedures in CVT.

### Supplementary Information

Below is the link to the electronic supplementary material.

Supplementary file1 (DOCX 1.18 MB)

## Data Availability

The data that support the findings of this study are available from the corresponding author on reasonable request.
