# Supplementary material for: Clinical value of neuroimaging indicators of intracranial hypertension in patients with cerebral venous thrombosis
Source: Neuroradiology. 2024 Apr 27;66(7):1161–76. doi: 10.1007/s00234-024-03363-6 (PMC11150314; doi:10.1007/s00234-024-03363-6)
Supplement: Supplementary file 1 — Supplementary file1 (DOCX 1.18 MB) [file 234_2024_3363_MOESM1_ESM.docx]

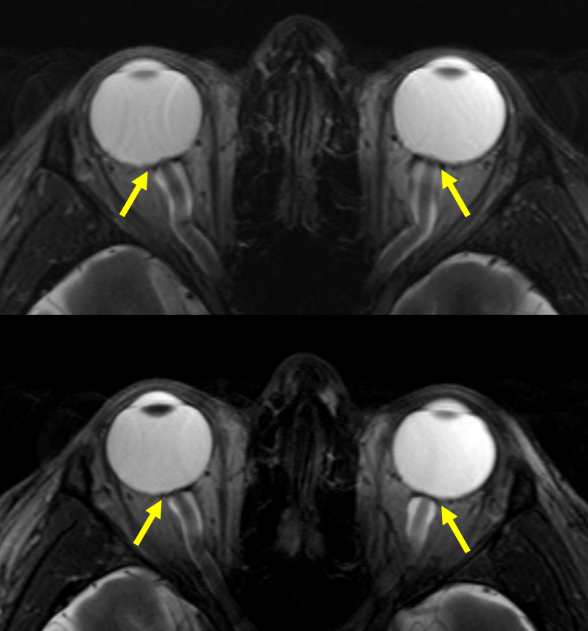


**Supplementary Fig. 1.** Ocular bulb flattening (patient P7) depicted on axial T2 weighted turbospin echo imaging (yellow arrows) at baseline (upper picture) and regression at follow-up (lower picture). Also note the optic disc protrusion at baseline and the normal shaped optic nerve head at follow-up.


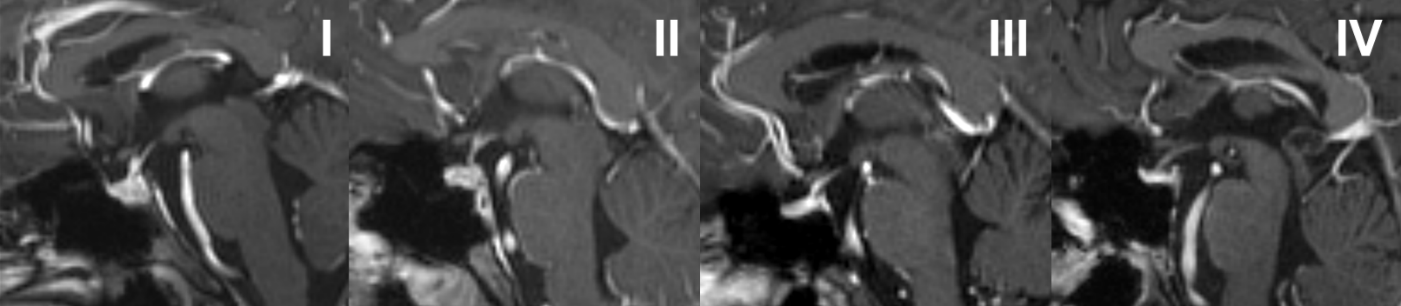


**Supplementary Fig. 2.** Pituitary grading according to Yuh et al. [16] referring to the reduction of visible pituitary tissue within the sella turcica. Normal aspect (grade I); mild superior concavity (grade II, less than 1/3 of sella height); moderate concavity (grade III, between 1/3 and 2/3 of sella height); severe concavity (grade IV, more than 2/3 of sella height). Grade V (empty sella, not shown) was not present in our CVT cohort.


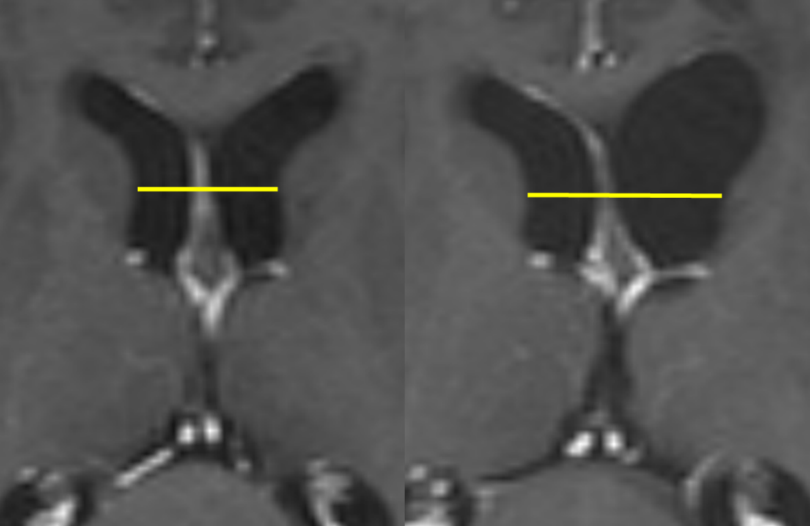


**Supplementary Fig. 3.** Lateral ventricle size measurement as described by Dong et al. [14] in patient P6, showing increasing dilatation between baseline (left) and follow-up (right).


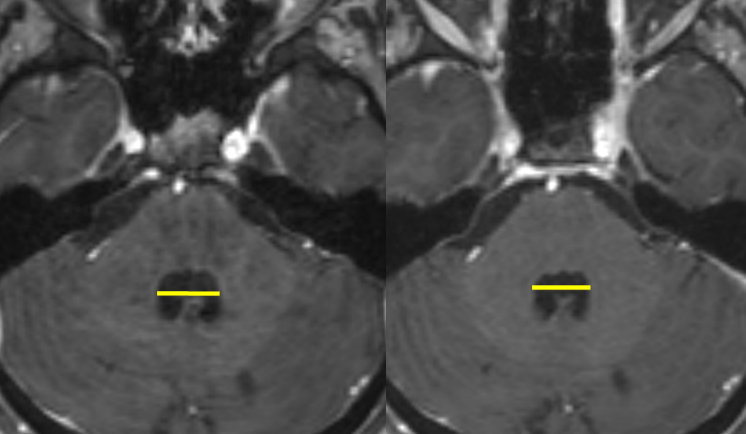


**Supplementary Fig. 4.** Fourth ventricle size measurement as described by Dong et al. [14] in patient P6, showing slight reduction between baseline (left) and follow-up (right).

**
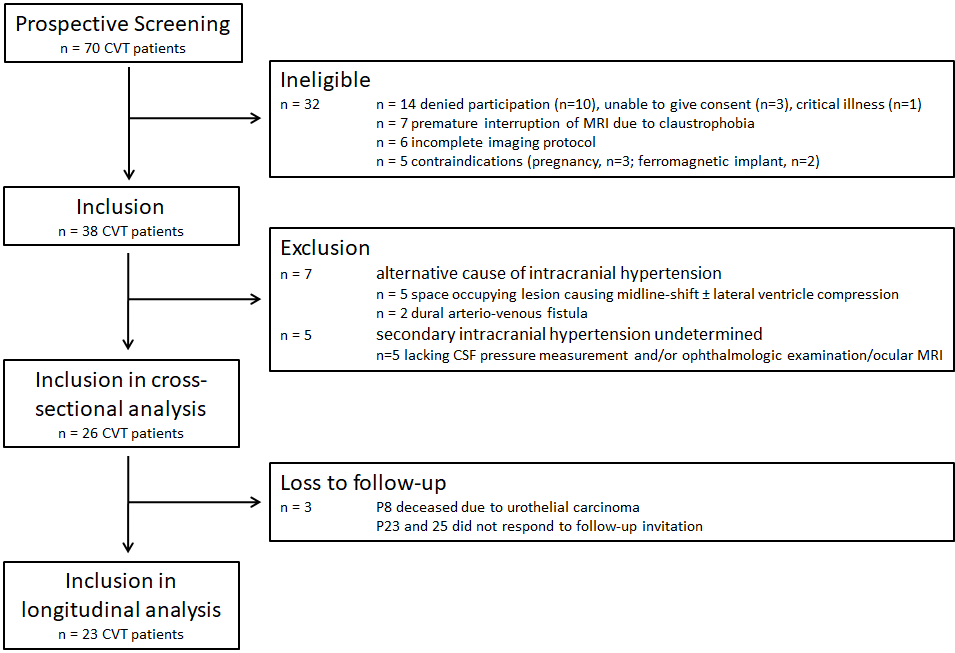
**

**Supplementary Fig. 5** Patient selection

**
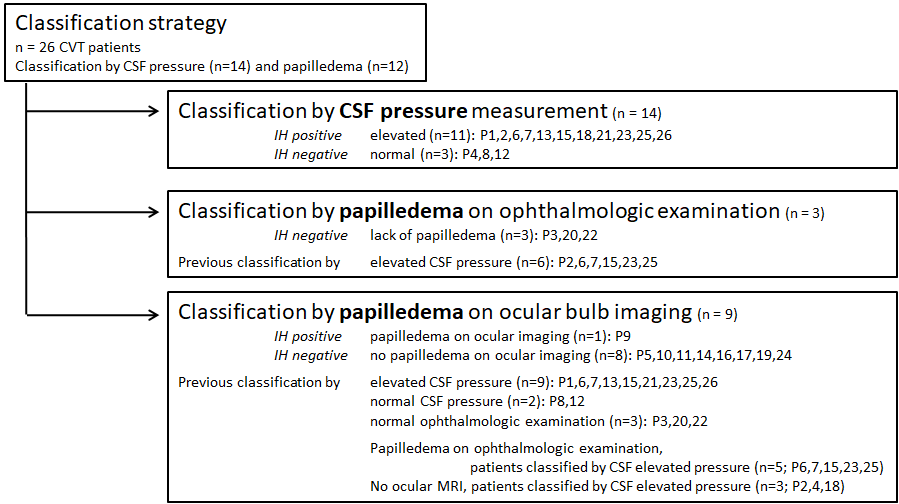
**

**Supplementary Fig. 6** Classification strategy of CVT patients (IH classification based on cerebro-spinal fluid (CSF) pressure ≥ 25cm H_2_O and/or papilledema on optic coherence tomography or optic disc protrusion on ocular MRI [3]).

**
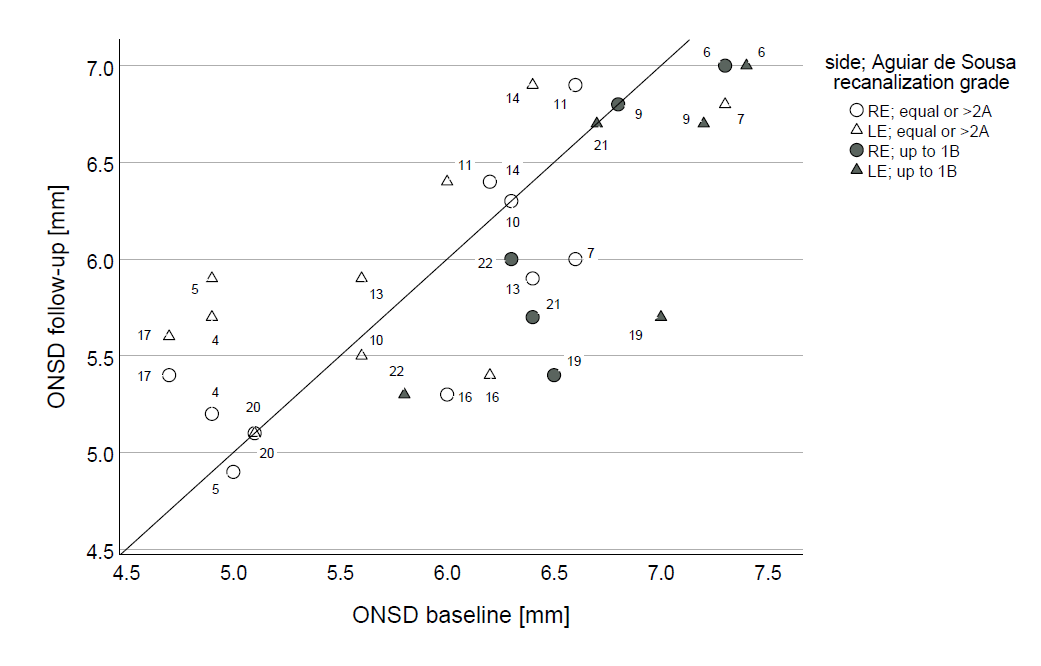
**

**Supplementary Fig. 7**. Distribution of individual participants optic nerve sheath diameter (ONSD) by side and classified by the extent of recanalization (Aguiar de Sousa ≥2A [25] or Qureshi III [26]). Filled triangles and circles depict measurements in patients lacking recanalization degrees Qureshi III or Aguiar de Sousa ≥2A, and empty symbols represent patients showing recanalization to at least this extent. Circles (right eyes) and triangles (left eyes) located on the diagonal line were stable at follow-up compared with baseline. Compared with baseline, ONSD above the line increased until follow-up, ONSD below the line decreased between the two measurements

| **Sequence type** | **Parameter** |
| --- | --- |
| 3D FLAIR SPACE | TR/TE, 5000 ms/388 ms; TI, 1800 ms; voxel size, 1 x 1 x 1 mm^3^; FOV, 250 x 250 x 176 mm^3^; sagittal slab; TA, 7 min 37 sec |
| SWI | TR/TE, 28 ms/20 ms; voxel size, 0.8 x 0.7 x 1.2 mm^3^; flip angle, 15°; FOV, 230 x 172 x 127 mm^3^; axial slices; TA, 6 min 0 sec |
| TOF MRA | TR/TE 20 ms/4.83 ms; voxel size, 1 × 1 × 2.5mm^3^; flip angle, 60°; FOV, 250 × 250 × 119mm^3^, coronal slab; TA, 3 min 34 sec |
| 3D T1w MPRAGE | TR/TE, 1390 ms/2.15 ms; TI, 800 ms; voxel size, 1 x 1 x 1 mm^3^; flip angle, 15°; FOV, 256 x 256 x 240 mm^3^; sagittal slab; TA, 3 min 26 sec |
| T2w HASTE | TR/TE, 1700 ms/129 ms; number of excitations, 1; bandwidth, 196 Hz/pixel; FOV, 19 x 16 cm^2^; Matrix, 448 x 378; phase encoding direction left to right; nominal spatial resolution, 0.42 x 0.42 mm^2^; slice thickness, 2 mm; TA, 1.7 sec per coronal slice |

**Supplementary Table 1**. MRI protocol. FLAIR SPACE, fluid attenuated inversion recovery/sampling perfection with application-optimized contrasts using different flip angle evolution; FOV, Field of view; HASTE, T2-weighted half-Fourier acquisition single-shot turbo-spin echo; MPRAGE, contrast-enhanced magnetization-prepared rapid acquisition with gradient echo; SWI, susceptibility weighted imaging; TOF MRA, time of flight MR angiography; TR/TE, repetition time/echo time; TI inversion time.

| Recanalization grade | | n (%) | 95% CI | Definition |
| --- | --- | --- | --- | --- |
| **Qureshi** | **Aguiar de Sousa** |  |  |  |
| - | 0 | 3 (14.3) | 0-31.8 | Persistent occlusion of all thrombosed vessels at baseline |
| I | - | 3 (14.3) | 0-31.8 | Anterograde flow through only part of the sinus with improved collateral flow or visualization of branches |
| IIA | 1B | 1 (4.8) | 0-15 | Combination of persistent occlusion(s) and complete recanalization(s), with/without partial recanalization(s) |
| IIB | 1A | 2 (9.5) | 0-23.8 | Combination of persistent occlusion(s) and partial recanalization(s), without any complete recanalization |
| III | - | 15 (71.4) | 52.4-90.5 | Complete recanalization of all occluded venous sinuses |
| - | 2A | 2 (9.5) | 0-23.8 | Partial recanalization of all thrombosed vessels, without any persistent occlusion |
| - | 2B | 6 (28.6) | 9.5-50 | Combination of partial and complete recanalization(s), without any persistent occlusion |
| - | 3 | 7 (33.3) | 13.6-55 | Complete recanalization of all previously thrombosed vessels |

**Supplementary Table 2**. Recanalization grading according to the classifications of Qureshi and Aguiar de Sousa. Follow-up venography was available in 21 patients (patients P15 and P18 refused contrast application at follow-up). One third showed complete recanalization and over 70% at least partial recanalization of all previously thrombosed vessels.

| **parameter** | **Aguiar de Sousa grade** | | | **Qureshi grade** | |
| --- | --- | --- | --- | --- | --- |
|  | Any recanalization  **p-value** | ≥ 2A  **p-value** | Complete recanalization  **p-value** | Any recanalization  **p-value** | III  **p-value** |
| ONSD size FU | 0.384 | 0.356 | 0.600 | 0.720 | 0.356 |
| ONSD difference baseline - FU | 0.173 | 0.077 | 0.610 | 0.287 | 0.077 |
| Fourth ventricle size FU | 1.0 | 0.852 | 0.231 | 0.466 | 0.852 |
| Fourth ventricle difference baseline - FU | 0.254 | 0.494 | 0.481 | 0.661 | 0.494 |
| Lateral ventricle size FU | 0.215 | 0.122 | 0.947 | 0.206 | 0.122 |
| Lateral ventricle difference baseline - FU | 0.055 | 0.097 | 0.383 | 0.059 | 0.097 |

**Supplementary Table 3.** Association between neuroimaging findings and recanalization grading (Kendalls’ tau).
